# Incident Gout: Risk of Death and Cause-Specific Mortality in Western Sweden: A Prospective, Controlled Inception Cohort Study

**DOI:** 10.3389/fmed.2022.802856

**Published:** 2022-02-24

**Authors:** Mats Dehlin, Tatiana Zverkova Sandström, Lennart TH Jacobsson

**Affiliations:** Department of Rheumatology and Inflammation Research, Institute of Medicine, Sahlgrenska Academy, University of Gothenburg, Gothenburg, Sweden

**Keywords:** gout, mortality, cause of death, dementia, case control study

## Abstract

**Background:**

Excess mortality in gout has been attributed to cardiovascular diseases (CVD). Considering the decline in CVD mortality in the general population, we wanted to evaluate overall mortality in gout and cause-specific contributions to mortality beyond CVD and temporal trends.

**Methods:**

All incident cases of gout between 2006 and 2015 in western Sweden and 5 population controls per case matched for age, sex, and county were identified. Comorbidities were identified for 5 years preceding the index date. Follow-up ended at death, migration, or end of study on December 2017. Effect of gout on death risk was calculated using COX regression on the whole population and stratified by sex, adjusted for demographics, and comorbidities. Death incidence rates were compared between the two time periods, 2006–2010 and 2011–2015.

**Results:**

We identified 22,055 cases of incident gout and 98,946 controls, median age (Q1, Q3) 69–68 (57, 79/56, 78) years and 67.6–66.5% males. Except for dementia, all comorbidities were significantly more common at baseline among gout cases. Overall, the risk for death in incident gout was neither increased overall nor in men, but women had a 10% elevated risk. In adjusted models for cause-specific mortality, death from CVD, renal disease, and digestive system diseases were significantly increased in the total gout population while death from dementia, cancer, and lung diseases were significantly decreased. There were no significant differences in overall incident death rate ratios between cases and controls in the two time periods examined.

**Conclusions:**

An increased risk for CVD, renal disease, and diseases of the digestive system in patients with gout highlights the importance of addressing CVD risk factors in gout management. Gout was associated with reduced mortality from dementia, which may have implications on urate lowering therapy and possible effects on dementia risk.

## Introduction

Gout is the most common inflammatory joint disease in the world (1). It is caused by oversaturation of urate, hyperuricemia, leading to deposition and crystallization of urate in joints and tendons which triggers a pronounced immunological reaction in susceptible individuals resulting in severe pain and excessive inflammation (2). Acute treatment of gout is available and effective, examples hereof are nonsteroidal antiinflammatory drugs (NSAID), corticosteroids, and colchicine. Prophylactic treatment with urate lowering therapy (ULT) is cheap, available, and offers a “cure” for the disease, but in spite of this it is suboptimally used in Sweden and the world (3, 4). Gout is closely associated with a number of comorbidities. Some of these give rise to hyperuricemia, thus increasing the risk of gout, examples hereof are renal disease and obesity (5). Other comorbidities, such as cardiovascular disease (CVD) and cancer have no clear causal connection to gout (6, 7). Gout and hyperuricemia have been associated with a decreased risk for neurodegenerative diseases, such as dementia and Parkinson's (8, 9), although there are conflicting results (10–12). To further complicate matters, CVD is a risk factor for dementia (13).

Gout has been associated with an increased overall mortality (14), and increased mortality is due to CVD (15, 16), kidney disease, endocrine, and metabolic disease (17, 18). Comorbidities probably explain the main part of the increased mortality seen in patients with gout, but other factors have been investigated. A low level of education has been shown to be related not only to premature mortality in the general population (19) but also in patients with gout (20). Presence of tophi and high urate, markers of more severe gout disease, have also been shown to increase the risk for cardiovascular death (21, 22). However, treatment with ULT has not so far been shown to have a protective effect on mortality (23). Women represent a minority of gout patients and have to a lesser extent been studied, but the mortality risk has been shown to be increased in both sexes although the risk increment was higher in women (17, 18, 24). Studies of the effects of gout disease duration on mortality risk have shown contradictory results (18, 25).

A recent register study from Sweden including all inhabitants aged ≥ 18 years in Skåne showed a 17% increased risk of all-cause mortality. For cause-specific mortality, the study showed an increased risk for death from renal disease, diseases of the digestive system, CVD, and infections, whereas the risk was decreased for death from dementia (26).

Life expectancy is increasing worldwide as well as in Sweden. This is to a large extent explained by a decrease in CVD mortality due to improved cardiovascular health care (27). Decreased mortality in rheumatoid arthritis over the last decades has been shown and this may also be explained by a decrease in CVD mortality (28), whereas a similar trend has not been observed in patients with gout over the same time period (29). One explanation for this may be an increasing impact of nonCVD comorbidities on mortality in patients with gout. Following this, it is important to increase the knowledge and provide contemporary results regarding both all-cause and cause-specific mortality in gout. This may also have implications for future treatment recommendations.

In the present study, our aims were to determine the relative risks in incident gout patients in comparison to the general population 1) for overall death 2) for cause-specific mortality, and 3) to examine possible temporal trends.

## Materials and Methods

### Data Sources

#### The Western Swedish Health Care Register (VEGA)

The register contains information about all healthcare contacts in in- and outpatient secondary care clinics and in primary care in the Western Swedish Health Care Region (WSHCR). Date of contact and primary and secondary diagnoses given by the treating physician, according to the Swedish version of the International Statistical Classification of Diseases (ICD-10), are registered. VEGA was used to identify gout cases and to retrieve information about relevant comorbidities for cases and controls. For gout cases, the date of the first gout diagnosis was used as the index date.

#### The Swedish Census Register

The census register holds demographic information about all registered inhabitants of Sweden, including date of death and emigration. The census register was used to identify up to 5 controls for each case, matched for age, sex, and place of residence on municipality level at the year of identification. Controls were assigned the same index date as their corresponding case.

#### The Longitudinal Integration Database for Health Insurance and Labor Market Studies (LISA)

The LISA register is administered by Statistics Sweden and holds annual registers on all individuals 16 years of age and older. From here data on education level, income, marital status, birthplace outside Sweden, and migration was retrieved.

#### The Swedish Prescribed Drug Register

All prescribed drugs and the date for dispensation by Swedish pharmacies are recorded in the Swedish Prescribed Drug register. Prescription of ULT (ATC-code M04AA and M04AB) was retrieved from here. The register has been available since July 1, 2005.

#### The Cause of Death Register

The Swedish Cause of Death Register contains information from death certificates recorded according to the ICD-10 system (30). The Swedish Cause of Death Register follows recommendations from the World Health Organization for identifying the underlying cause of death (30). It has been shown to have high validity with respect to main diagnostic categories (31, 32). We identified the underlying cause of death for all study subjects who died between January 1, 2006 and December 31, 2017.

#### The Cancer Register

The Swedish Cancer Register, maintained by the National Board of Health and Welfare, was founded in 1958 and covers the whole population of Sweden. All data on cancer comorbidity was retrieved from here.

### Study Population

The Swedish healthcare system is tax-funded and offers universal access. Gout is typically diagnosed and treated in a primary care setting. All inhabitants aged ≥18 years in the WSHCR from January 1, 2006 to December 31, 2015 constituted the study population. The population of WSHCR is approximately 1.6 million (20% of the total population of Sweden) (33), and the region is considered to be representative for Sweden with regard to health status, healthcare seeking, and socioeconomics (33, 34).

To select individuals most likely to have new-onset gout, we included individuals, aged ≥18 years, with a diagnosis of gout in the VEGA-register during 2006–2015. The VEGA database was then searched for gout diagnoses back to January 1, 2000, and individuals with a prior diagnosis of gout were excluded. All included cases thus have a period free from gout diagnosis of at least 5 years before their index date. Gout diagnosis was defined by the presence of an ICD-10 code for gout (M10), registered at a visit to a physician in the VEGA-database. We excluded individuals with dispensation of ULT (ATC-code M04AA and M04AB) prior to the first gout diagnosis. Up to 5 controls per case matched for age, year, and municipality at the year of gout diagnosis were identified in the Swedish Census Register. Controls with a prescription of ULT were excluded. The case definition has been previously validated by means of record review, which showed a high validity of ICD-10 codes for gout in the VEGA-database (35). Follow-up started from the index date and ended at the date of death, relocation outside of WSHCR, or the end of the study (December 31, 2017), whichever occurred first.

### Outcome

In this prospective and controlled inception cohort study, the outcomes were all-cause mortality and cause-specific mortality defined as: cardiovascular disease, renal disease, dementia, infections, diabetes, diseases of the digestive system, lung diseases, neoplasms, and other. For definitions, (see Supplementary Table 1). We identified the underlying cause of death for all study subjects who died between the start of follow-up and the end of follow-up, which was defined as the first of death, emigration, or December 31, 2017. To examine temporal trends of death in incident gout, we divided the study period in two, 2006–2010 and 2011–2015. Thereafter, we calculated incident death rate ratios in total, and subdivided into CVD and non-CVD causes of death between cases and controls in the two time periods.

### Confounders

The following confounders were recorded in the year of the index date for cases and controls: sex, marital status, income, education, and whether the person was born outside of Sweden. The following comorbidities: alcohol-related disorders, hypertension, ischemic heart disease, heart failure, cerebrovascular disease, diabetes mellitus, dyslipidemia, obesity, chronic kidney disease, dementia, and lung diseases were considered as possible confounders and they were identified in the VEGA database for at least 5 years prior to the year of the index date. Data on neoplasms were retrieved from the Swedish Cancer Register. For definitions, (see Supplementary Table 1).

### Statistics

All data management and analyses were performed in SAS 9.4 and R 4.0.3. Baseline features were described as count (percent) of categorical data and as mean [standard deviation (SD)] along with median (25th and 75th percentiles) of continuous data. Incidence rate (IR) of total mortality and cause-specific mortality were calculated per 1,000 person-year under the assumption of Poisson distribution. Cause-specific mortality was treated throughout the study as follows: the cause of death in question was considered as an event and the other causes of death were censured. Then 95% confidence intervals of incidence rate ratios (IRR) were calculated under the assumption of normal distribution for the natural logarithm of IRR. Hazard ratios (HR) were computed with Cox proportion hazard model. We used two models for adjustment in the COX regression. Model 1 adjusted for sex and age at baseline and Model 2 adjusted for sex and age at baseline, marital status, income, education, born outside of Sweden, alcohol-related disorders, hypertension, ischemic heart disease, heart failure, cerebrovascular disease, diabetes mellitus, dyslipidemia, obesity, chronic kidney disease, dementia, lung diseases, and any neoplasm. In the COX regression analysis, we adjusted for violation of proportionality assumption (when present) by including significant time-interactions in the final models. To examine the possible impact of competing causes of death on our results, we used the Fine and Gray competing risk regression model (36).

## Results

### Baseline Characteristics

From the total adult population of WSHCR between 2006 and 2015, with a yearly average population of 1,264,150 individuals, we identified 22,055 cases of incident gout and 98,946 controls, for details (see Table 1). After certain exclusions (Table 1) the median age (Q1, Q3) was 69 (57, 79) years in the gout cohort compared with 68 (56, 78) in the controls and 67.6% were men in the gout cases compared with 66.5% in the controls, (Table 2). Median annual income, level of education, and being born in Sweden was lower or less frequent in the gout subjects, (Table 2). All identified comorbidities were significantly more common among the gout cases with the exception of dementia that was more frequent in the non-gout control subjects, (Table 2). When stratified for sex, findings were similar, except for a higher age in incident female gout cases, median (Q1, Q3) 74 (62, 83) years compared to males, 67 (55, 76), (Supplementary Table 2).

**Table 1:** Flowchart of the study population.

	**Cases**	**Controls**
Identified in VEGA	56,771	
Identified in The Swedish Census Register		110,081
Re-used/missing personal identification number	−78	0
=	56,693	110,081
No diagnosis of gout	−6,199	N/A
=	50,494	110,081
Not WSHCR resident	−117	0
=	50,377	110,081
Gout diagnosis before 2006	−11,249	−69
=	39,128	110,012
Gout diagnosis after 2015	−9,932	N/A
=	29,196	110,012
First emigration date before diagnosis	−216	−28
=	28,980	109,984
First immigration date 30 days after diagnosis	−43	−176
=	28,937	109,808
ULT (M04AA, M04AB) within 5 years before diagnosis	−6,860	−1,432
=	22,077	108,376
Age at diagnosis <18 years	−22	
=	22,055	108,376
Died before Index date	0	−291
	22,055	108,085
Control more than once	N/A	−9,139
		
Study population	22,055	98,946

*WSHCR, Western Sweden Health Care Region; VGR, western sweden health care region. N/A, not applicable*.

**Table 2:** Baseline characteristics in gout cases and general population controls.

	**Gout cases, *n* = 22,055**	**Controls, *n* = 98,946**	***p*-value**
Age, years, mean (SD)	67.1 (15.3)	66.3 (15.4)	<0.0001
Age, years, median (Q1, Q3)	69 (57, 79)	68 (56, 78)	<0.0001
Male sex, n (%)	14,926 (67.6)	65,914 (66.5)	0.002
Annual income, EURO, median (Q1, Q3)	15,600 (12,040, 23,450)	16,230 (12,050, 24,760)	<0.0001
Education, n (%)			<0.0001
≤9 years	8,716 (39.6)	36,480 (36.9)	
10–12 years	8,938 (40.6)	38,459 (39.0)	
≥13 years	4,013 (18.2)	22,391 (22.7)	
Married, n (%)	11,718 (53.3)	52,694 (53.4)	0.8
Born outside Sweden, n (%)	3,216 (14.6)	13,611 (13.8)	0.001
**Comorbidities, n (%)**			
Alcohol related disorders	932 (4.2)	2,337 (2.4)	<0.0001
Hypertension	12,590 (57.1)	35,276 (35.7)	<0.0001
Ischemic heart disease	5,217 (23.7)	13,265 (13.3)	<0.0001
Heart failure	4,180 (19.0)	6,603 (6.7)	<0.0001
Cerebrovascular disease	2,269 (10.3)	7,568 (7.7)	<0.0001
Diabetes mellitus	3,965 (18.0)	10,550 (10.7)	<0.0001
Dyslipidemia	5,756 (26.1)	15,859 (16.0)	<0.0001
Obesity	1,033 (4.7)	1,642 (1.7)	<0.0001
Chronic kidney disease	964 (4.4)	822 (0.8)	<0.0001
Dementia	545 (2.5)	3,909 (4.0)	<0.0001
Lung diseases	3,181 (14.4)	9,242 (9.3)	<0.0001
Neoplasm	2,608 (11.8)	10,760 (10.9)	<0.0001
**Follow-up time years:**			
*Mean (SD)*	5.5 (3.0)	5.6 (3.0)	0.02
*Median (Q1; Q3)*	5.2 (3.2; 7.7)	5.3 (3.2; 7.7)	0.04

*SD, standard deviation; Q, quartile*.

### Death Incidence Rates

**Figure 1:**
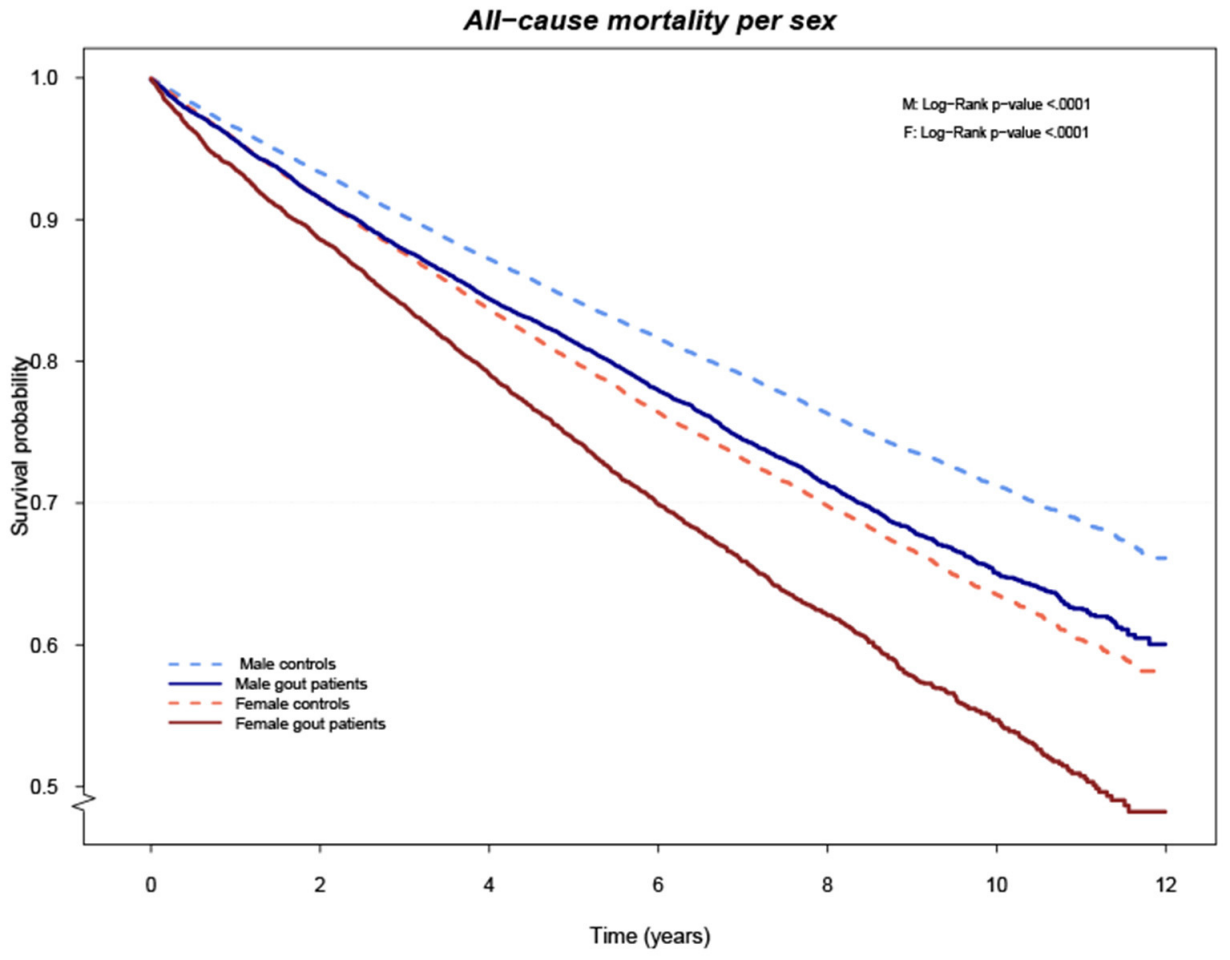
Cumulative risk of death overall comparing cases and controls stratified by gender.

After a mean (SD) follow-up time of 5.5 (3.0) years for gout cases and 5.6 (3.0) years for controls, the survival curves differed significantly between cases and controls, in both men and women (Figure 1). We identified 5,817 (26.4%) deaths among the gout cases, resulting in an IR of 47.7 per 1,000 person-years, which was significantly higher compared to the 20,753 (21.0%) deaths, IR 37.6 per 1,000 person-years, among controls, with an IRR (95% CI) of 1.27 (1.23–1.31), (Table 3). Overall, CVD was the major cause of death in both groups, although significantly more common in gout individuals with an IRR (95% CI) of 1.56 (1.50–1.63), (Table 3 and Supplementary Figure 1). Death by renal disease, infections, diabetes, diseases of the digestive system, lung diseases, and others were all significantly more common in gout subjects compared to controls, (Table 3 and Supplementary Figure 1). The opposite was found for death caused by dementia, IR (95% CI) 2.01 (0.50–8.01) in gout subjects compared to 3.96 (1.48–10.60) in controls, IRR 0.51 (0.45–0.58), (Table 3 and Supplementary Figure 1).

**Table 3:** Number of deaths and incidence rates, overall and cause-specific, in cases and controls.

**Cause of death, n (%)**	**Gout cases, *n* = 22,055**	**Incidence rate per 1,000 person-years (95% CI)**	**Controls, *n* = 98,946**	**Incidence rate per 1000 person-years (95% CI)**	**Incidence rate ratio (95% CI)**
Total deaths	5,817 (26.4)	47,74 (35.95–63.40)	20,753 (21.0)	37.60 (27.32–51.76)	1.27 (1.23–1.31)
Cardiovascular disease	2,905 (49.9)	23.84 (15.96–35.62)	8,406 (40.5)	15.23 (9.22–25.17)	1.56 (1.50–1.63)
Renal diseases	102 (1.8)	0.84 (0.10–7.13)	171 (0.8)	0.31 (0.01–10.48)	2.70 (2.11–3.45)
Dementia	245 (4.2)	2.01 (0.50–8.01)	2,185 (10.5)	3.96 (1.48–10.60)	0.51 (0.45–0.58)
Infections	362 (6.2)	2.97 (0.95–9.26)	1,193 (5.8)	2.16 (0.57–8.20)	1.37 (1.2–1.55)
Diabetes	202 (3.5)	1.66 (0.36–7.60)	500 (2.4)	0.91 (0.12–7.10)	1.83 (1.55–2.15)
Diseases of the digestive system	183 (3.2)	1.50 (0.30–7.43)	534 (2.6)	0.97 (0.13–7.10)	1.55 (1.31–1.84)
Lung diseases	254 (4.4)	2.08 (0.54–8.10)	912 (4.4)	1.65 (0.36–7.59)	1.26 (1.10–1.45)
Neoplasms	1,006 (17.3)	8.26 (4.17–16.33)	4,650 (22.4)	8.43 (4.29–16.55)	0.98 (0.9–1.05)
Other	558 (9.6)	4.58 (1.83–11.44)	2,202 (10.6)	3.99 (1.50–10.64)	1.15 (1.05–1.26)

*CI, confidence intervals*.

### Death Incidence Rates and Sensitivity Analysis

We have also performed a sensitivity analysis on IRRs for total deaths comparing gout cases with controls stratified by age groups, in all subjects, and by sex, which showed significantly higher IRRs in gout cases in all age groups in total and divided by sex, with the exception of women aged 18–40, where no significant difference was seen, (see Supplementary Table 3). Furthermore, the IRRs were consistently higher for women but attenuated with increasing age.

### Risk of Death

All-cause mortality was significantly increased in the gout group with a HR of 1.24 (95% CI 1.21–1.28, *p* < 0.0001) when adjusting for age and sex, but was substantially attenuated to 1.03 (95% CI 1.00–1.06, *p*-value 0.079) in the fully adjusted model, (Table 4). Stratified by sex, the relative impact of gout on all-cause mortality was higher and significantly increased only in women compared to men in the fully adjusted models, HR 1.10 (95% CI 1.05–1.15) vs. 0.99 (95% CI 0.95–1.03), respectively, (Table 4).

**Table 4:** Total and cause-specific mortality, overall and stratified by sex, in gout cases compared to controls.

**Causes of death**	**Cause-specific mortality**								**Cause-specific mortality. Fine-gray method**			
	**Overall**				**Male**		**Female**		**Overall**		**Male**	**Female**
	**Model 1**	***p*-value**	**Model 2**	***p*-value**	**Model 2**	***p*-value**	**Model 2**	***p*-value**	**Model 1**	**Model 2**	**Model 2**	**Model 2**
Cardiovascular disease	1.54 (1.47–1.60)	<0.0001	1.13 (1.08–1.18)	<0.0001	1.07 (1.01–1.14)	0.02	1.23 (1.15–1.32)	<0.0001	1.54 (1.48–1.61)	1.17 (1.12–1.23)	1.13 (1.06–1.20)	1.25 (1.16–1.34)
Renal diseases	2.62 (2.05–3.35)	<0.0001	1.53 (1.17–2.01)	0.002	1.60 (1.16–2.21)	0.004	1.32 (0.79–2.20)	0.3	2.47 (1.93–3.15)	1.55 (1.15–2.08)	1.64 (1.16–2.31)	1.30 (0.72–2.36)
Dementia	0.40 (0.32–0.50)	<0.0001	0.65 (0.57–0.75)	<0.0001	0.65 (0.54–0.79)	<0.0001	0.67 (0.55–0.81)	<0.0001	0.45 (0.40–0.52)	0.68 (0.59–0.78)	0.70 (0.57–0.84)	0.67 (0.55–0.82)
Infections	1.34 (1.19–1.51)	<0.0001	1.10 (0.97–1.25)	0.1	1.06 (0.91–1.25)	0.4	1.21 (0.98–1.48)	0.07	1.25 (1.11–1.40)	1.13 (1.00–1.28)	1.10 (0.94–1.28)	1.19 (0.97–1.46)
Diabetes	1.79 (1.52–2.11)	<0.0001	0.94 (0.79–1.12)	0.5	0.89 (0.71–1.11)	0.3	1.04 (0.79–1.37)	0.8	1.69 (1.43–1.99)	0.98 (0.82–1.17)	0.96 (0.76–1.20)	1.02 (0.77–1.35)
Diseases of the digestive system	1.52 (1.29–1.80)	<0.0001	1.27 (1.06–1.52)	0.01	1.18 (0.94–1.49)	0.2	1.43 (1.08–1.89)	0.01	1.44 (1.22–1.71)	1.30 (1.09–1.56)	1.24 (0.98–1.55)	1.41 (1.06–1.87)
Lung diseases	1.24 (1.08–1.42)	0.003	0.86 (0.74–0.99)	0.04	0.81 (0.67–0.98)	0.03	0.89 (0.71–1.12)	0.3	1.17 (1.02–1.34)	0.86 (0.75–1.00)	0.83 (0.68–1.01)	0.89 (0.71–1.11)
Neoplasms	0.86 (0.77–0.97)	0.01	0.90 (0.84–0.97)	0.003	0.87 (0.80–0.94)	0.0009	0.98 (0.86–1.11)	0.8	0.90 (0.84–0.96)	0.91 (0.84–0.97)	0.88 (0.80–0.96)	0.98 (0.86–1.11)
Other	1.12 (1.02–1.23)	0.01	1.03 (0.93–1.14)	0.6	1.02 (0.90–1.15)	0.8	1.05 (0.89–1.24)	0.6	1.06 (0.96–1.16)	1.05 (0.95–1.16)	1.06 (0.94–1.19)	1.03 (0.87–1.22)
All-cause mortality	1.24 (1.21–1.28)	<0.0001	1.03 (1.00–1.06)	0.079	0.99 (0.95–1.03)	0.5	1.10 (1.05–1.15)	0.0002				

*Results are presented as, age/sex- and fully adjusted hazard ratios (95% confidence intervals), both without and with adjustment for competing risks (Fine and Grey method), using Cox proportional hazard models*.

*CI, confidence intervals*.

*Model 1: adjusted for sex and age at baseline*.

*Model 2: adjusted for sex and age at baseline, marital status, income, education, born outside of Sweden, alcohol related disorders, hypertension, ischemic heart disease, heart failure, cerebrovascular disease, diabetes mellitus, dyslipidemia, obesity, chronic kidney disease, dementia, lung diseases, any neoplasm*.

Death attributed to CVD was significantly higher in gout cases overall, HR 1.13 (95% CI 1.08–1.18), for both men and women, (Table 4). Death due to renal disease and diseases of the digestive system were significantly higher in gout overall, HR 1.53 (95% CI 1.17–2.01) and 1.27 (95% CI 1.06–1.52), respectively, but when stratified for sex, renal disease was only significantly increased in men with gout, HR 1.60 (95% CI 1.16–2.21), while death due to digestive diseases only was significantly increased in women, HR 1.43 (95% CI 1.08–1.89), (Table 4). Death attributed to dementia was on the other hand significantly lower in gout cases overall 0.65 (95% CI 0.57–0.75), both for men 0.65 (95% CI 0.54–0.79) and women 0.67 (95% CI 0.55–0.81), (Table 4). Furthermore, this reduced risk of death in gout cases was seen for all of the four most common specific causes of death categorized under dementia: unspecified dementia, Alzheimer's disease, vascular dementia, and Parkinson's disease, (Supplementary Table 4). On the other hand, dementia as a comorbidity at baseline was relatively more common in the control subjects in two of the three largest death cause groups, CVD, and infections, but not in cancer compared to cases; this was true for both overall and stratified by sex, (Supplementary Table 5). Furthermore, dementia at baseline was much more common in female cases and controls, but this may be explained by their higher baseline age. A lower risk of death in gout patients was also seen for death caused by lung diseases and neoplasms overall, HR 0.86 (95% CI 0.74–0.99) and 0.90 (95% CI 0.84–0.97), respectively, but only significantly in men when stratifying for sex, HR 0.81 (95% CI 0.67–0.98) and 0.87 (95% CI 0.80–0.94), respectively, (Table 4). For information on specific diagnosis of death in each category, (see Supplementary Table 4).

### Risk of Death and Sensitivity Analysis

In the sensitivity analysis, where possible impact of competing causes of death on our results was evaluated with the method proposed by Fine and Gray (36), all point estimates were in the same direction and of similar magnitude, (see Table 4).

### Temporal Trends

There were no significant differences in incident death rate ratios in the total, and subdivided into CVD and non-CVD cause of death, between cases and controls in the two time periods examined, 2006–2010 vs. 2011–2015, (see Supplementary Table 6).

## Discussion

In this study, we found significantly increased mortality in patients with gout. After adjusting for age, sex, birthplace outside Sweden, and relevant comorbidities, the overall risk for death in incident gout was not increased neither overall nor in men, whereas women had a 10% elevated risk. However, the gout patients had a significantly increased risk for death caused by CVD, renal disease, and diseases of the digestive system and a significantly decreased risk for death caused by dementia, lung diseases, and neoplasms in fully adjusted models, with similar results for men and women. We found no significant temporal trends of death rate between the first and the last part of the study period.

### Death Overall

Overall death has previously been reported to be increased in patients with gout (14, 17, 25, 26), a finding that has been attributed to typical gout characteristics and comorbidities (14). In the health professionals' follow-up study, Choi et al. found an increased risk for overall death of 28% in male patients with a history of gout but without coronary heart disease at baseline compared to non-gout male individuals (25). In Taiwan, more than 6,000 gout patients were followed for 8 years through 2008 and compared to the national population and the all-cause standardized mortality ratio (95% CI) was 1.29 (1.21–1.37) for men and 1.70 (1.53–1.89) for women (17). In a recent population-based register study from southern Sweden, gout was associated with 17% increased hazard of all-cause mortality, 23% in women, and 15% in men (26).

### Cardiovascular Disease

As expected, CVD was the main cause of death for patients with gout in our study, which is in line with previous studies (15–18, 22). Not only is CVD morbidity frequent at the time of first gout diagnosis, but in addition gout is treated with potentially cardiotoxic acute medicines such as NSAIDs or corticosteroids over time. This emphasizes the need for CVD screening in gout patients and also an increased use of ULT to diminish the need for acute treatment. Furthermore, gout is characterized by episodes of severe inflammation, which in addition may increase the risk for CVD. This is supported by results from the CARES trial in 2017 where treatment with the anti-inflammatory, anti-gout, and interleukin-1 inhibitor canakinumab decreased the risk of recurrent cardiovascular events (37).

### Renal Disease

Death caused by renal disease was significantly increased in the gout patients in line with previous studies (17, 18). Decreased renal secretion of urate by genetic predisposition and/or kidney disease is a major cause of hyperuricemia, which in a certain proportion will lead to gout (38). At the time of diagnosis, the gout patients in this study had an increased occurrence of multiple risk factors for kidney damage such as hypertension, diabetes, and obesity. In addition, they also have an increased use of acute medication for gout attacks with potentially nephrotoxic NSAIDs. With appropriate and increased use of ULT, such need for acute treatment could be diminished. The possible negative effect of gout on kidney function is still unresolved and requires further research (39).

### Infections

In the present study, death due to infections was not increased in gout patients. In contrast, the risk was found increased in a recent population-based register study from southern Sweden (26) and in a cohort study from Taiwan comparing 6,000 (1,400 female) gout patients with the general population (17). A population-based cohort study using data from the UK Clinical Practice Research Datalink that included 130 000 gout patients and 250 000 controls from 1987 to 2014 did not find an increased risk of infection-related mortality (40) in gout patients, whereas a Dutch prospective cohort study with crystal proven gout showed an increased risk for death due to infectious disease as well as cancer and CVD (22). The frequency of gout increases with age and so do comorbidities. A recent study from the US by Singh et al. have shown an increase in serious infections leading to hospitalization over the last two decades in gout patients. The most common infections seen were pneumonia and sepsis, with age and comorbidities as the major identified contributing factors (41). In the present study, pneumonia and sepsis were the major infectious causes of death in both cases and controls. These findings give weight to the EULAR guidelines regarding vaccination from 2019 where it is advised to consider pneumococcal vaccination for the majority of patients with inflammatory rheumatic diseases (42).

### Dementia

In our study, gout protected against death by dementia, which was also shown in the aforementioned population-based register study from southern Sweden (26). Register studies have shown a decreased risk for dementia in gout individuals (43) and metaanalysis of crosssectional studies has identified a protective effect of (increasing) urate on the risk for Alzheimer's dementia (8) while longitudinal studies show conflicting results (10–12). In the present study, we lack data on urate levels. Obesity is strongly associated with both gout and hyperuricemia and was more common in gout subjects in our study. The obesity paradox refers to the dual, age-dependent, effects on dementia, where overweight and obesity in middle age are associated with an increased future dementia risk in old age but when examined later in life higher BMI is linked to better cognition and decreased mortality (44), which may have influenced the results in our study. It should be noted that our definition of obesity at baseline likely underestimated the occurrence and that we lacked data specifically on BMI. Our findings are however supported by the observation that diagnosed dementia was less common in gout cases vs. controls already at the start of follow-up. The possible protective effect of hyperuricemia on dementia development needs to be further examined and related to the treatment target for urate suggested by current ACR and EULAR treatment guidelines (45, 46).

### Lung Disease

Gout has not been found to be associated with smoking (47, 48), although exposure to air pollution or inorganic dust may have a role in gout pathogenesis (49, 50). Nevertheless, lung diseases were a more common comorbidity in the gout population of our study. In spite of this, we saw a significant protective effect of gout on death from lung diseases in men, which have not been shown in earlier studies (17, 26). In the current study, we have no specific data on smoking exposure. However, in 2017 we performed a questionnaire study in 800 gout patients in the same region of western Sweden where we, for both men and women, found similar frequencies for current smokers but significantly higher frequencies for former smokers among gout patients compared to controls (51). The two most common lung-related causes of death in the current study in both cases and controls were chronic obstructive pulmonary disease and interstitial pulmonary disease, but small numbers limit further analysis.

### Cancer

Prevalence of neoplasms was higher at the time of first gout diagnosis compared to non-gout controls in our study. In spite of this, we saw a protective effect of gout on death attributed to cancer which have not been shown in earlier population-based studies (17, 26). Gout is characterized by hyperuricemia which has potent antioxidant properties, possibly protecting against cancer. However, gout is also closely associated with insulin resistance, obesity, and increased alcohol intake, all of which are well-known carcinogenic risk factors. An increased risk for some types of cancers has been shown in gout patients, particularly urinary tract cancers, cancers of the digestive system, and lung cancer (7). In the current study, the four major causes of death by cancer were cancer of the prostate, lung, pancreas, and large intestine/colon in both cases and controls. This mirrors the incidence and mortality of cancer in Sweden (52).

### Digestive System

The increased risk of death from diseases of the digestive system in our study is supported by previous findings (17, 26). Diagnosis of alcohol-related disorders were significantly higher at the time of first gout diagnosis compared to the controls in our study. This may, at least partly, explain the increased mortality from alcoholic liver disease, which was the only cause-specific death of the digestive system that was significantly increased in the gout cases in the study.

### Diabetes

Diabetes was one of the more common comorbidities at baseline in the gout patients of our study. In spite of this, we could not find an elevated risk of death from diabetes, which contrasts some previous findings (17, 53).

### General Risk Factors

Higher income is associated with decreased mortality in the Nordic countries (54), and lower level of education is a risk factor for increased mortality in the general population as well as in gout patients (19, 20). The gout patients in our study had lower income and educational level compared with controls and were more often born outside Sweden. Increased mortality risk due to CVD and cancer has been shown in Swedish immigrants (55, 56). The mortality risk increment in women compared to men with gout must be interpreted in the context of factors such as a lower overall mortality in women, higher age of gout onset, and consequently more comorbidities at the start and during follow-up (5).

There are some limitations to this study. First, as in all register studies there is a risk for misclassification of diagnosis. However, we have in a previous study found that the gout diagnosis has high validity (57). Second, obesity and alcohol abuse was in the study only defined by ICD-10 codes at baseline, and we lack data specifically on BMI and alcohol intake, at baseline and during follow-up, which will lead to an underestimation of these exposures. Third, we lack data on smoking. Fourth, urate may have a role in many of the mortality causes in the study, not least dementia, but we lack data on urate levels. Fifth, ULT and colchine use may have affected the urate levels and the inflammatory response, but we lack data on these medications.

There are also some strengths to this study. The study is population-based with a large sample size, which minimizes the risk for selection bias. Data on cause of death, cancer, and other comorbidities were collected from three different registers which all have almost complete coverage of the population. Second, the study was performed on incident cases of gout minimizing the risk for survival bias. Finally, sensitivity analyses taking into account competing causes of death showed similar results.

## Conclusions

To conclude, in this study we found an increased risk for death caused by CVD, renal disease, and diseases of the digestive system highlighting the importance of addressing not only CVD risk factors but also infections, diseases of the digestive system, and renal diseases in the management of patients with gout. Furthermore, gout was associated with reduced mortality from dementia, and these findings call for further study, not least because of possible effects of ULT on the risk of dementia.

## Data Availability Statement

The raw data supporting the conclusions of this article will be made available by the authors, without undue reservation.

## Ethics Statement

The studies involving human participants were reviewed and approved by Ethical Review Board of Gothenburg, Sweden. Written informed consent for participation was not required for this study in accordance with the national legislation and the institutional requirements.

## Author Contributions

MD contributed to the conception and design of the study, as well as managing and interpretation of data, and drafting and revising the manuscript. TS contributed to interpretation of data, was responsible for statistical work, and participated in drafting and revising the manuscript. LJ contributed to the conception and design of the study, as well as interpretation of data, and drafting and revising the manuscript. All authors have contributed substantially in the process of completing this study and had full access to the data and approved the manuscript and agree to be accountable.

## Funding

Funding for the study was received from the following sources: Reumatikerfonden and Gothenburg University.

## Conflict of Interest

The authors declare that the research was conducted in the absence of any commercial or financial relationships that could be construed as a potential conflict of interest.

## Publisher's Note

All claims expressed in this article are solely those of the authors and do not necessarily represent those of their affiliated organizations, or those of the publisher, the editors and the reviewers. Any product that may be evaluated in this article, or claim that may be made by its manufacturer, is not guaranteed or endorsed by the publisher.
