# Supplementary material for: Incident Gout: Risk of Death and Cause-Specific Mortality in Western Sweden: A Prospective, Controlled Inception Cohort Study
Source: Front Med (Lausanne). 2022 Feb 24;9:802856. doi: 10.3389/fmed.2022.802856 (PMC8907510; doi:10.3389/fmed.2022.802856)
Supplement: Supplementary file 1 [file Table_1.docx]

Supplementary Table 1 Definitions of comorbidities and grouped causes of death by ICD-10 code

| Variable | ICD-10 code |
| --- | --- |
| Comorbidity | |
| Alcohol related disorders | F10 |
| Hypertension | I10 |
| Ischemic heart disease | I20-I25 |
| Heart failure | I50 |
| Cerebrovascular disease | I60–I69 |
| Diabetes mellitus | E10–E14 |
| Dyslipidemia | E78 |
| Obesity | E66.9, E66P |
| Chronic kidney disease | N18 |
| Dementia | F01, F03, G20 |
| Lung diseases | J43–J45 |
| Neoplasm | C, D43, D48, D38, D47, D41, D37 |
| Cause of death groups | |
| Cardiovascular disease | I00-I99 |
| Renal diseases | N009-N059, N111-N289, Q61.3 |
| Dementia, ICD-10 code | F01, F03, G20, G30 |
| Infections | A, B, J00-J22, J85-J86, L089, N10, N39, M00-M001 |
| Diabetes | E10-E14 |
| Diseases of the digestive system | K |
| Lung diseases | J30-J84, J90-J99 |
| Neoplasms | C, D |
| Other | E035 - E079, E162 – E889, F051 - F99, G001 – G14, G214 - G258, G35 - G98, H669, L021 - L039, L108 - L984, M0519 - M954, N300 – N359, N40 - N939, P159, Q02 - Q602, Q631 - Q969, R02 - R999, V021 - V959, W00 - W87, X00 - X99, Y00 - Y891, |
