# Supplementary material for: Incident Gout: Risk of Death and Cause-Specific Mortality in Western Sweden: A Prospective, Controlled Inception Cohort Study
Source: Front Med (Lausanne). 2022 Feb 24;9:802856. doi: 10.3389/fmed.2022.802856 (PMC8907510; doi:10.3389/fmed.2022.802856)
Supplement: Supplementary file 2 [file Table_2.docx]

Suppl Table 2 Baseline characteristics in gout cases and general population controls, stratified by sex

|  | Gout, male n= 14 914 | Control, male n= 65 855 | p-value | Gout, female n= 7 141 | Control, female n= 33 091 | p-value |
| --- | --- | --- | --- | --- | --- | --- |
| Age, years, mean (SD) | 65.1 (15.0) | 64.2 (15.0) | <.0001 | 71.1 (15.0) | 70.6 (15.1) | 0.006 |
| Age, years, median (Q1, Q3) | 67 (55, 76) | 66 (54, 76) | <.0001 | 74 (62, 83) | 73 (61, 82) | 0.0044 |
| Annual income, EURO, median (Q1, Q3) | 17 770 (13 240, 23 440) | 18 550 (13 240, 27 440) | <.0001 | 12 780 (10 260, 16 680) | 13 210 (10 480, 18 510) | <.0001 |
| Education, n (%) |  |  | <.0001 |  |  | <.0001 |
| ≤9 years | 5 425 (36.5) | 22 802 (34.7) |  | 3 291 (46.2) | 13 678 (41.4) |  |
| 10–12 years | 6 331 (42.6) | 26 596 (40.5) |  | 2 607 (36.6) | 11 863 (35.9) |  |
| ≥13 years | 2 905 (19.5) | 15 479 (23.6) |  | 1 108 (15.5) | 6 912 (20.9) |  |
| Married, n (%) | 8 688 (58.4) | 38 240 (58.2) | 0.6 | 3 030 (42.5) | 14 454 (43.8) | 0.051 |
| Born outside Sweden, n (%) | 2 104 (14.1) | 9 221 (14.0) | 0.7 | 1 112 (15.6) | 4 390 (13.3) | <.0001 |
| Comorbidities, n (%) |  |  |  |  |  |  |
| Alcohol related disorders | 789 (5.3) | 2 017 (3.1) | <.0001 | 320 (1.0) | 143 (0.4) | <.0001 |
| Hypertension | 7 955 (53.3) | 21 287 (32.3) | <.0001 | 4 635 (64.9) | 13 989 (42.3) | <.0001 |
| Ischemic heart disease | 3 512 (23.6) | 9 193 (14.0) | <.0001 | 1 705 (23.9) | 3 972 (12.0) | <.0001 |
| Heart failure | 2 526 (16.9) | 4 109 (6.2) | <.0001 | 1 654 (23.2) | 2 494 (7.5) | <.0001 |
| Cerebrovascular disease | 1 455 (9.8) | 4 815 (7.3) | <.0001 | 814 (11.4) | 2 753 (8.3) | <.0001 |
| Diabetes mellitus | 2 460 (16.5) | 7 336 (11.1) | <.0001 | 1 505 (21.1) | 3 214 (9.7) | <.0001 |
| Dyslipidemia | 3 847 (25.8) | 10 362 (15.7) | <.0001 | 1 909 (26.7) | 5 497 (16.6) | <.0001 |
| Obesity | 642 (4.3) | 1 049 (1.6) | <.0001 | 391 (5.5) | 593 (1.8) | <.0001 |
| Chronic kidney disease | 664 (4.5) | 622 (0.9) | <.0001 | 300 (4.2) | 200 (0.6) | <.0001 |
| Dementia | 287 (1.9) | 2 038 (3.1) | <.0001 | 258 (3.6) | 1 871 (5.7) | <.0001 |
| Lung diseases | 1 839 (12.3) | 5 504 (8.4) | <.0001 | 1 342 (18.8) | 3 738 (11.3) | <.0001 |
| Neoplasm | 1 744 (11.7) | 7 137 (10.8) | 0.003 | 846 (12.1) | 3 623 (11.0) | 0.005 |
