# Supplementary material for: Incident Gout: Risk of Death and Cause-Specific Mortality in Western Sweden: A Prospective, Controlled Inception Cohort Study
Source: Front Med (Lausanne). 2022 Feb 24;9:802856. doi: 10.3389/fmed.2022.802856 (PMC8907510; doi:10.3389/fmed.2022.802856)
Supplement: Supplementary file 3 [file Table_3.docx]

Suppl table 3, Incidence rate ratio for total deaths comparing gout cases with controls stratified by age groups, in all subjects and by gender,

|  | Incidence rate ratio, 95% CI | | |
| --- | --- | --- | --- |
| Age group | All subjects | Male | Female |
| 18-40 | 2.76 (1.59-4.78) | 3.28 (1.70-6.32) | 1.88 (0.67-5.27) |
| 41-50 | 2.03 (1.55-2.65) | 1.68 (1.22-2.31) | 3.52 (2.11-5.89) |
| 51-60 | 1.50 (1.28-1.75) | 1.38 (1.16-1.64) | 1.99 (1.44-2.76) |
| 61-70 | 1.35 (1.24-1.47) | 1.24 (1.12-1.36) | 1.76 (1.49-2.09) |
| 71-80 | 1.25 (1.19-1.32) | 1.17 (1.09-1.25) | 1.43 (1.30-1.58) |
| 81+ | 1.20 (1.15-1.24) | 1.16 (1.10-1.22) | 1.23 (1.16-1.30) |

CI = confidence intervals
