# Supplementary material for: Incident Gout: Risk of Death and Cause-Specific Mortality in Western Sweden: A Prospective, Controlled Inception Cohort Study
Source: Front Med (Lausanne). 2022 Feb 24;9:802856. doi: 10.3389/fmed.2022.802856 (PMC8907510; doi:10.3389/fmed.2022.802856)
Supplement: Supplementary file 4 [file Table_4.docx]

Supplementary Table 4, Number of deaths, incidence rates and incidence rate ratios in gout cases and general population controls, by causes of death in more detail

| ICD-10 code,  Specific cause of death | Gout cases,  n = 22 055, n (%) | Incidence rate per 1000 person-years (95% CI) | Controls,  n= 98 946, n (%) | Incidence rate per 1000 person-years (95% CI) | Incidence rate ratio (95% CI) |
| --- | --- | --- | --- | --- | --- |
| Cardiovascular disease | 2 905 (49.9) | 23.84 (15.96-35.62) | 8 406 (40.5) | 15.23 (9.22-25.17) | 1.56 (1.50-1.63) |
| I25, Chronic  ischaemic heart disease | 760 (13.1) | 36.84 (26.67-50.88) | 1709 (8.2) | 23.05 (15.33-34.68) | 1.60 (1.47-1.74) |
| I21, Acute  myocardial infarction | 462 (7.9) | 22.40 (14.80-33.89) | 1535 (7.4) | 20.71 (13.46-31.85) | 1.08 (0.97-1.20) |
| I50, Heart failure | 306 (5.3) | 14.83 (8.92-24.67) | 850 (4.1) | 11.47 (6.43-20.45) | 1.29 (1.14-1.47) |
| I48, Atrial  fibrillation and flutter | 277 (4.8) | 13.43 (7.86-22.92) | 656 (3.2) | 8.85 (4.58-17.10) | 1.52 (1.32-1.75) |
| I64, Stroke | 115 (2.0) | 5.57 (2.43-12.79) | 522 (2.5) | 7.04 (3.36-14.74) | 0.79 (0.65 -0.97) |
|  |  |  |  |  |  |
| Renal diseases | 102 (1.8) | 0.84 (0.10-7.13) | 171 (0.8) | 0.31 (0.01-10.48) | 2.70 (2.11-3.45) |
| N18, Chronic kidney disease | 51 (0.9) | 2.47 (0.71-8.60) | 57 (0.3) | 0.77 (0.08-7.19) | 3.22 (2.20-4.69) |
| N19, Unspecified  kidney failure | 25 (0.4) | 1.21 (0.20-7.19) | 62 (0.3) | 0.84 (0.10-7.13) | 1.45 (0.91-2.31) |
| N17, Acute renal failure | 10 (0.2) | 0.48 (0.03-8.09) | 11 (0.1) | 0.15 (0.00-24.05) | 3.27 (1.39-7.69) |
|  |  |  |  |  |  |
| Dementia | 245 (4.2) | 2.01 (0.50-8.01) | 2 185 (10.5) | 3.96 (1.48-10.60) | 0.51 (0.45-0.58) |
| F03, Unspecified dementia | 154 (2.6) | 7.47 (3.64-15.30) | 1278 (6.2) | 17.24 (10.75-27.64) | 0.43 (0.37-0.51) |
| G30, Alzheimer disease | 40 (0.7) | 1.94 (0.47-7.92) | 463 (2.2) | 6.25 (2.85-13.68) | 0.31 (0.22-0.43) |
| F01, Vascular dementia | 39 (0.7) | 1.89 (0.45-7.86) | 269 (1.3) | 3.63 (1.30-10.15) | 0.52 (0.37-0.73) |
| G20, Parkinson disease | 12 (0.2) | 0.58 (0.04-7.60) | 175 (0.8) | 2.36 (0.66-8.45) | 0.25 (0.14-0.44) |
|  |  |  |  |  |  |
| Infections | 362 (6.2) | 2.97 (0.95-9.26) | 1 193 (5.8) | 2.16 (0.57-8.20) | 1.37 (1.2-1.55) |
| J18, Pneumonia | 137 (2.4) | 6.64 (3.10-14.21) | 516 (2.5) | 6.96 (3.31-14.63) | 0.95 (0.79-1.15) |
| A41, Sepsis | 90 (1.5) | 4.36 (1.71-11.15) | 238 (1.1) | 3.21 (1.08-9.59) | 1.36 (1.07-1.73) |
| B99, Infection NOS | 29 (0.5) | 1.41 (0.27-7.34) | 113 (0.5) | 1.52 (0.31-7.46) | 0.92 (0.61-1.39) |
| N39,Urinary tract infection | 21 (0.4) | 1.02 (0.15-7.10) | 72 (0.3) | 0.97 (0.13-7.10) | 1.05 (0.64-1.70) |
| A09, Gastroenteritis | 11 (0.2) | 0.53 (0.04-7.81) | 46 (0.2) | 0.62 (0.05-7.47) | 0.86 (0.45-1.66) |
|  |  |  |  |  |  |
| Diabetes | 202 (3.5) | 1.66 (0.36-7.60) | 500 (2.4) | 0.91 (0.12-7.10) | 1.83 (1.55-2.15) |
| E14, Unspecified  diabetes mellitus | 140 (2.4) | 6.79 (3.20-14.40) | 319 (1.5) | 4.30 (1.67-11.07) | 1.58 (1.29-1.92) |
| E11, Type 2 diabetes mellitus | 54 (0.9) | 2.62 (0.78-8.79) | 167 (0.8) | 2.25 (0.61-8.31) | 1.16 (0.85-1.58) |
| E10, Type 1 diabetes mellitus | 8 (0.1) | 0.39 (0.02-9.03) | 14 (0.1) | 0.19 (0.00-17.17) | 2.05 (0.86-4.89) |
|  |  |  |  |  |  |
| Diseases of the  digestive system | 183 (3.2) | 1.50 (0.30-7.43) | 534 (2.6) | 0.97 (0.13-7.10) | 1.55 (1.31-1.84) |
| K92, Diseases of the digestive system NOS | 27 (0.5) | 1.31 (0.24-7.26) | 63 (0.3) | 0.85 (0.10-7.12) | 1.54 (0.98-2.42) |
| K70, Alcoholic liver disease | 23 (0.4) | 1.11 (0.17-7.13) | 48 (0.2) | 0.65 (0.06-7.40) | 1.72 (1.05-2.83) |
| K56, Intestinal obstruction | 17 (0.3) | 0.82 (0.10-7.14) | 61 (0.3) | 0.82 (0.09-7.14) | 1.00 (0.59-1.71) |
| K55, Vascular disorder  of the intestine | 13 (0.2) | 0.63 (0.05-7.44) | 33 (0.2) | 0.45 (0.02-8.40) | 1.42 (0.75-2.69) |
| K74, Hepatic fibrosis | 10 (0.2) | 0.48 (0.03-8.09) | 42 (0.2) | 0.57 (0.04-7.66) | 0.86 (0.43-1.71) |
|  |  |  |  |  |  |
| Lung diseases | 254 (4.4) | 2.08 (0.54-8.10) | 912 (4.4) | 1.65 (0.36-7.59) | 1.26 (1.10-1.45) |
| J44, Chronic obstructive pulmonary disease | 175 (3.0) | 8.48 (4.33-16.63) | 608 (2.9) | 8.20 (4.14-16.26) | 1.03 (0.87-1.22) |
| J84, Interstitial  pulmonary disease | 36 (0.6) | 1.75 (0.40-7.69) | 109 (0.5) | 1.47 (0.29-7.40) | 1.19 (0.81-1.73) |
| J45, Asthma | 11 (0.2) | 0.53 (0.04-7.81) | 30 (0.1) | 0.40 (0.02-8.81) | 1.32 (0.66-2.63) |
|  |  |  |  |  |  |
| Neoplasms | 1 006 (17.3) | 8.26 (4.17-16.33) | 4 650 (22.4) | 8.43 (4.29-16.55) | 0.98 (0.9-1.05) |
| C34, Lung cancer | 145 (2.5) | 7.03 (3.36-14.72) | 666 (3.2) | 8.98 (4.67-17.28) | 0.78 (0.65-0.94) |
| C61, Prostate cancer | 139 (2.4) | 6.74 (3.17-14.34) | 823 (4.0) | 11.10 (6.16-19.99) | 0.61 (0.51-0.73) |
| C25, Pancreas cancer | 85 (1.5) | 4.12 (1.57-10.82) | 314 (1.5) | 4.24 (1.63-10.98) | 0.97 (0.77-1.24) |
| C80, Cancer NOS | 63 (1.1) | 3.05 (0.99-9.37) | 240 (1.2) | 3.24 (1.09-9.62) | 0.94 (0.71-1.24) |
| C18, Colon cancer | 62 (1.1) | 3.01 (0.97-9.31) | 417 (2.0) | 5.63 (2.46-12.85) | 0.53 (0.41-0.70) |
|  |  |  |  |  |  |
| Other | 558 (9.6) | 4.58 (1.83-11.44) | 2 202 (10.6) | 3.99 (1.50-10.64) | 1.15 (1.05-1.26) |
| R99, Other ill-defined and unspecified causes of death | 78 (1.3) | 3.78 (1.38-10.36) | 295 (1.4) | 3.98 (1.49-10.63) | 0.95 (0.74-1.22) |
| X59, Exposure to  unspecified factor | 58 (1.0) | 2.81 (0.87-9.05) | 233 (1.1) | 3.14 (1.04-9.49) | 0.89 (0.67-1.19) |
| W19, Unspecified fall | 50 (0.9) | 2.42 (0.69-8.54) | 157 (0.8) | 2.12 (0.55-8.14) | 1.14 (0.83-1.57) |
| R54, Senility | 30 (0.5) | 1.45 (0.29-7.39) | 214 (1.0) | 2.89 (0.91-9.15) | 0.50 (0.34-0.74) |
| D46, Myelodysplastic syndromes | 16 (0.3) | 0.78 (0.08-7.18) | 35 (0.2) | 0.47 (0.03-8.18) | 1.64 (0.91-2.97) |

CI = confidence intervals, NOS = Not Otherwise Specified,
