# Supplementary material for: Incident Gout: Risk of Death and Cause-Specific Mortality in Western Sweden: A Prospective, Controlled Inception Cohort Study
Source: Front Med (Lausanne). 2022 Feb 24;9:802856. doi: 10.3389/fmed.2022.802856 (PMC8907510; doi:10.3389/fmed.2022.802856)
Supplement: Supplementary file 5 [file Table_5.docx]

Suppl table 5 Dementia as a diagnosed comorbidity at baseline in all subjects, in subjects dead due to CVD, neoplasm or infection during follow-up, overall and by sex

|  | All subjects | | | Male | | | Female | | |
| --- | --- | --- | --- | --- | --- | --- | --- | --- | --- |
| Dementia at baseline | Gout cases | Controls | Gout % / Controls % | Gout cases | Controls | Gout % / Controls % | Gout cases | Controls | Gout % / Controls % |
| All subjects, % | 2.5 | 4.0 | 0.63 | 1.9 | 3.1 | 0.61 | 3.6 | 5.7 | 0.63 |
| Dead due to CVD, % | 6.3 | 12.9 | 0.49 | 5.4 | 11.4 | 0.47 | 7.5 | 15.0 | 0.50 |
| Dead due to neoplasm, % | 2.5 | 3.7 | 0.68 | 2.3 | 3.7 | 0.62 | 2.8 | 3.7 | 0.76 |
| Dead due to infection, % | 6.9 | 14.5 | 0.48 | 7.6 | 13.8 | 0.55 | 5.8 | 15.8 | 0.37 |
