# Supplementary material for: Incident Gout: Risk of Death and Cause-Specific Mortality in Western Sweden: A Prospective, Controlled Inception Cohort Study
Source: Front Med (Lausanne). 2022 Feb 24;9:802856. doi: 10.3389/fmed.2022.802856 (PMC8907510; doi:10.3389/fmed.2022.802856)
Supplement: Supplementary file 6 [file Table_6.docx]

Suppl Table 6, Death incidence rate per 1000 person years and incidence rate ratios in two time periods for incident gout cases and matched controls

|  | 2006-2010 | | | | 2011-2015 | | | | Incidence Rate Ratio  Case / Control  (95% CI) | |
| --- | --- | --- | --- | --- | --- | --- | --- | --- | --- | --- |
| Cause of death | N (%) | | Incidence rate per 1000 person years (95 CI) | | N (%) | | Incidence rate per 1000 person years (95 CI) | | 2006-2010 | 2011-2015 |
|  | Case, n= 9733 (44.1) | Control, n= 43560 (44.0) | Case | Control | Case, n= 12322 (55.9) | Control, n= 55386 (56.0) | Case | Control |  |  |
| Total death | 3 555 (36.5) | 12 878 (29.6) | 49.35  (37.34-65.24) | 39.48  (28.90-53.93) | 2 262 (18.4) | 7 926 () | 45.46  (33.99-60.80) | 34.89  (25.04-48.62) | 1.25  (1.20-130) | 1.30  (1.24-1.36) |
| CVD death | 1 778 (50.0) | 5 296 (41.1) | 24.68  (16.64-36.62) | 16.24  (9.98-26.41) | 1 127 (49.8) | 3 129 (39.5) | 22.59  (14.96-34.12) | 13.78  (8.13-23.36) | 1.52  (1.44-1.60) | 1.64 (1.53-1.76) |
| Non CVD death | 1 777 (50.0) | 7 582 (58.9) | 24.67  (16.63-36.61) | 23.25  (15.48-34.90) | 1 135 (50.2) | 4 797 (60.5) | 22.87  (15.18-34.45) | 21.11  (13.78-32.34) | 1.06  (1.01-1.12) | 1.10 (1.01-1.15) |

CI = confidence intervals
