# Supplementary figures and images for: Incident Gout: Risk of Death and Cause-Specific Mortality in Western Sweden: A Prospective, Controlled Inception Cohort Study
Source: Front Med (Lausanne). 2022 Feb 24;9:802856. doi: 10.3389/fmed.2022.802856 (PMC8907510; doi:10.3389/fmed.2022.802856)

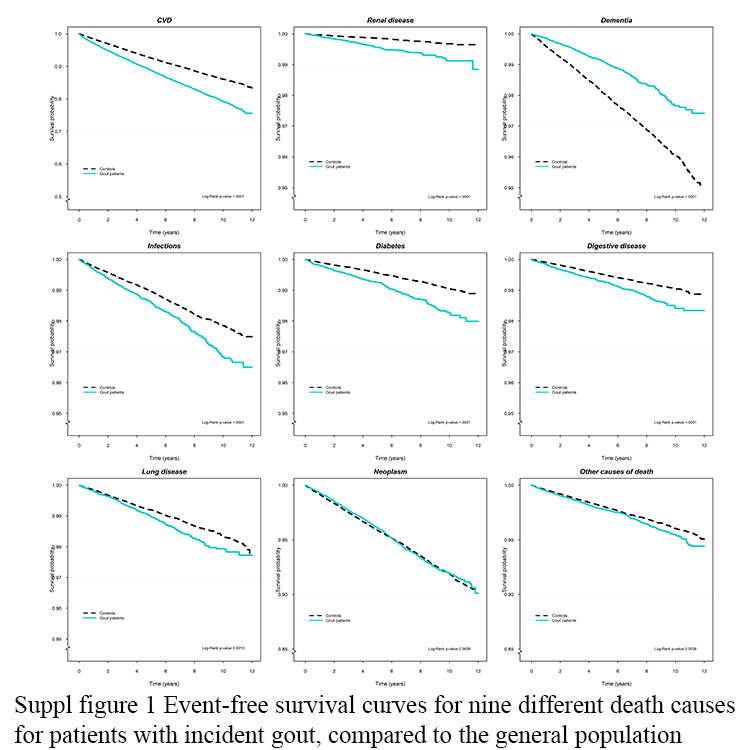

Supplement: Supplementary file 7 [file Image_1.TIF]
